# Clinical features of patients with Legionnaires disease showing initial clinical improvement but radiological deterioration: A retrospective single-center analysis

**DOI:** 10.1097/MD.0000000000028402

**Published:** 2021-12-23

**Authors:** Jinyeong Kim, Sunghee Park, Eunmi Yang, Haein Kim, Hyeonji Seo, Hyemin Chung, Jiwon Jung, Min Jae Kim, Yong Pil Chong, Sang-Oh Lee, Sang-Ho Choi, Yang Soo Kim, Sung-Han Kim

**Affiliations:** a Department of Infectious Diseases, Asan Medical Center, University of Ulsan College of Medicine, Seoul, South Korea; b Division of Infectious Disease, Department of Internal Medicine, Hanyang University Guri Hospital, South Korea.

**Keywords:** *Legionella*, Legionnaires disease, lung disease, pneumonia, radiography

## Abstract

Patients with Legionnaires disease occasionally experience initial clinical improvement but radiological progression. However, data on this issue are so far limited. The aim of this study was to investigate changes in chest radiograph findings in patients with Legionnaires disease who showed initial clinical improvement and to identify risk factors and outcomes in these patients.

All patients diagnosed with Legionnaires disease at a tertiary hospital in South Korea between March 2011 and May 2020 were retrospectively enrolled. Legionnaires disease was defined as abnormal chest radiographs accompanied by a positive finding on at least one of the following tests: urinary antigen test, sputum *Legionella* polymerase chain reaction, and sputum *Legionella* culture. Clinical improvement was defined as defervescence and decreased C-reactive protein level. Clinical and radiological records were reviewed on treatment days 7 and 14 and at discharge. We describe the characteristics of patients with clinical improvement but radiological deterioration on treatment for Legionnaires disease and compared them with patients with initial clinical improvement and stable or resolving chest radiograph findings.

Of 140 patients with Legionnaires disease, 33 (24%) showed initial clinical deterioration, while the remaining 107 (76%) showed initial clinical improvement on day 7. The latter 107 patients were analyzed in this study; 22 (21%) showed radiological progression despite the clinical improvement. Risk factors for these patients were a high pneumonia severity index score and the use of mechanical ventilation. Mortality did not significantly differ between those with initial clinical improvement but radiological deterioration and those with both initial clinical and radiological improvement (28% vs 12%, *P* = .49).

About one-fifth of patients with Legionnaires disease, especially those who had a high pneumonia severity index score and underwent mechanical ventilation, showed radiological deterioration despite of clinical improvement 1 week after appropriate treatment, while outcomes were not significantly worse in these patients. Therefore, our findings support that close monitoring without modification of antibiotics use is warranted in those who have clinical improvement regardless of radiologic findings.

## Abstract 2

Supplemental Digital Content is available in the text

## 1 Introduction

Legionnaires disease is a form of pneumonia caused by gram-negative bacteria, *Legionella* spp., which are found in freshwater environments worldwide and infect people via inhalation of contaminated aerosols.^[1,2]^ Incidence rates have been increasing annually, with 1.30 to 1.89 cases per 100,000 reported in surveillance studies in Europe^[3]^ and the United States^[4]^ in 2015. The worldwide burden of Legionnaires disease appears to be growing, as hospitalized patients with the disease are usually critically ill and often encounter respiratory failure following mechanical ventilation. A better understanding of the clinical features of hospitalized patients with Legionnaires disease may enable us to implement better management strategies. Patients with Legionnaires disease occasionally experience initial clinical improvement but radiological progression.^[5–7]^ However, data on this issue are limited, and the clinical meaning of the radiological discordance with respect to the clinical course of the disease is unknown. The aim of this study was to investigate changes in chest radiograph findings in patients with Legionnaires disease who showed initial clinical improvement and to identify risk factors and outcomes in these patients.

## 2 Methods

We retrospectively reviewed the medical records of all adult patients (age, ≥18 years) with Legionnaires disease between March 2011 and May 2020 at a 2700-bed tertiary care medical center in Seoul, South Korea. Legionnaires disease is characterized by a clinically compatible illness such as fever, myalgia, or respiratory symptoms, and at least one of the confirmatory laboratory criteria: positive urinary antigen test result, isolation of *Legionella* by culture, or positive detection by a validated polymerase chain reaction. Cases of Pontiac fever, characterized by a milder illness without pneumonia and extra-pulmonary legionellosis were excluded. If patients were suspected as pneumonia, beta-lactam with fluoroquinolone or fluoroquinolone monotherapy was recommended. When Legionnaires disease was suspected, routine diagnostic tests including urinary *Legionella* antigen and *Legionella* polymerase chain reaction from upper or lower respiratory samples were performed during the study period. Follow up chest radiograph and laboratory exam were assessed weekly at least until the hospital discharge or the discontinuation of antibiotic therapy. We selected and included the patients with Legionnaires disease who showed clinical improvement on treatment day 7. Clinical improvement was defined as defervescence and decreased C-reactive protein (CRP) level, as stable defervescence is one of the criteria used to define clinical stability^[8]^ and CRP can be a useful marker of treatment response.^[9–11]^ Defervescence was defined as decrease in body temperature under 37.8°C without antipyretic agents. Decreased CRP was defined if CRP level decreased to normal range (<0.5 mg/dL) or decreased comparing with pre-treatment peak level.

We collected the following information from the patients’ medical records: age, sex, comorbidities, initial laboratory findings, and serial chest radiograph findings. The severity of pneumonia was evaluated using the pneumonia severity index (PSI), with scores over 90 referred to as “high.”^[12]^ Chest radiographs were reviewed for changes on treatment days 7 and 14, as well as at discharge if patients were hospitalized. We based our analyses on 4 categories of radiographic change by using internationally standard nomenclature described by the Fleischner Society glossary^[13]^ and by referring to previous criteria for invasive fungal disease,^[14,15]^ as follows: “progression of disease,” defined as new or increased pulmonary parenchymal attenuation including consolidation, ground-glass opacity or pleural effusion, “stable disease,” defined as no significant changes of abnormal pre-existing or new findings, “partial response,” defined as decreased abnormal findings without newly developed lesions, and “complete response,” defined as the absence of any abnormal chest radiograph findings.

Categorical variables were compared using the chi-square test or Fisher exact test, as appropriate. Student *t* test was applied for continuous variables. All significance tests were two-tailed, and a *P* value <.05 was considered statistically significant. Data management and analysis were performed using IBM SPSS Statistics software version 21.0 (IBM Corp., Armonk, NY).

The study protocol was approved by the institutional review board of Asan Medical Center.

## 3 Results

**Figure 1:**
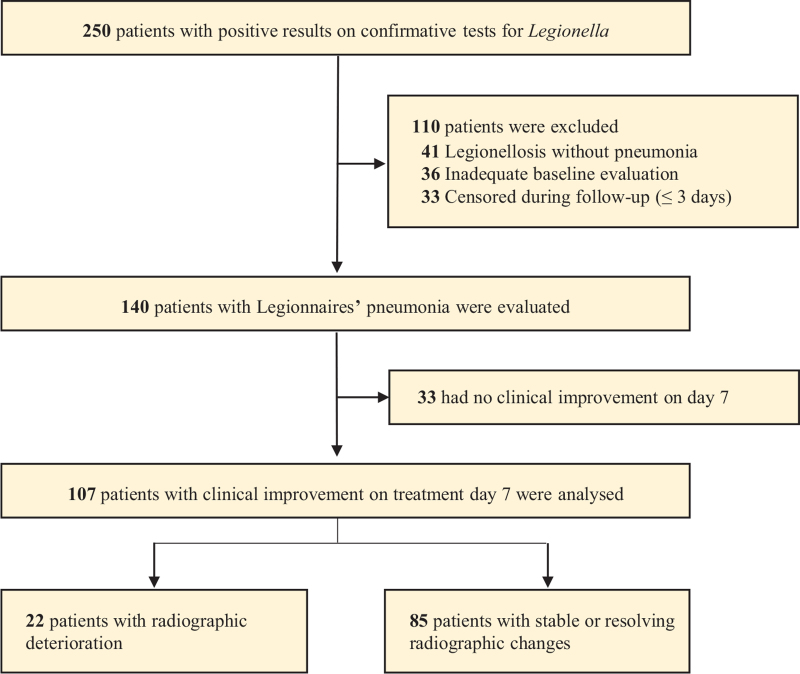
Study flowchart.

During the 10-year study period, 140 patients with Legionnaires disease were evaluated, with 33 (24%) showing initial clinical deterioration and the remaining 107 (76%) showing initial clinical improvement on day 7. The 107 patients with initial clinical improvement were analyzed in the current study (Fig. 1). The median age of the patients was 67 years, and males accounted for 71% of all patients. The baseline demographics and clinical characteristics according to initial radiological changes are shown in Table 1. Of the 107 patients with initial clinical improvement, 22 (21%) showed radiological deterioration on day 7, but then further radiological improvement between day 14 and the time of discharge (Table 2). Among the 22 patients who experienced initial clinical improvement but radiological progression at day 7, 13 patients (59%) had improvement in their radiographs within 2 weeks and 1 patient (5%) within 3 weeks. Six patients (27%) did not have further follow-up chest radiographs or medical records of their full hospital courses. Two patients (9%) expired with 2 weeks due to progressive pneumonia. The patients with initial clinical improvement who showed radiological deterioration had higher PSI scores (96% vs 78%, *P* = .007) and were more frequently treated with mechanical ventilators (46% vs 16%, *P* = .007) than those with initial clinical improvement and stable or resolving chest radiograph findings (Table 1). Concomitant organism was not significantly different between 2 groups for bacteria but respiratory virus (Table 1 and Table S1, Supplemental Digital Content, http://links.lww.com/MD2/A785). A total of 14 patients died from Legionnaires disease or other cause associated with their underlying diseases (Table S2, Supplemental Digital Content, http://links.lww.com/MD2/A786). Mortality did not significantly differ between those with initial clinical improvement but radiological deterioration and those showing both initial clinical and radiological improvement (28% vs 12%, *P* = .49). Multivariable analysis indicated that PSI score and mechanical ventilation were associated with radiological deterioration despite of initial clinical improvement (Table 3).

**Table 1:** Clinical characteristics of 107 patients with Legionnaires disease who showed initial clinical improvement according to radiologic changes observed on treatment day 7.

Variable	All patients (n = 107)	Chest radiographs showing deterioration (n = 22)	Stable or resolving chest radiograph findings (n = 85)	*P* value
Age, median, yrs	67 ± 13 (56–77)	69 ± 11 (58–79)	67 ± 14 (56–77)	.56
Male sex	76 (71)	17 (77)	59 (69)	.47
PSI score	119 ± 35 (96–140)	137 ± 32 (114–160)	114 ± 35 (93–133)	.007
PSI ≥ 91	87 (81)	21 (96)	66 (78)	.07
Hematologic malignancy	14 (13)	4 (18)	10 (12)	.48
Solid tumor	40 (37)	10 (46)	30 (35)	.38
Renal disease	25 (23)	4 (18)	21 (25)	.52
End-stage renal disease requiring hemodialysis	13 (12)	1 (5)	12 (14)	.30
Diabetes mellitus	24 (22)	4 (18)	20 (24)	.78
Chronic obstructive pulmonary disease	13 (12)	2 (9)	11 (13)	1.00
Mechanical ventilation	23 (22)	10 (46)	13 (16)	.007
Pleural effusion	36 (33)	10 (46)	26 (31)	.19
Laboratory findings				
WBC, number/μL	11,207 ± 8345 (4200–15,200)	12,081 ± 9901 (3525–17,725)	10,980 ± 7944 (5100–14,800)	.58
CRP, mg/dL	20.16 ± 10.78 (11.62–29.36)	21.69 ± 9.80 (14.37–31.11)	19.77 ± 11.03 (11.13–28.80)	.46
Creatinine, mg/dL	2.33 ± 3.72 (0.80–2.05)	1.73 ± 1.62 (0.80–2.10)	2.48 ± 4.08 (0.78–2.15)	.40
BUN, mg/dL	34 ± 26 (17–42)	33 ± 19 (20–41)	34 ± 28 (16–43)	.87
AST, IU/L	51 ± 80 (18–54)	40 ± 45 (16–45)	54 ± 87 (19–55)	.47
ALT, IU/L	40 ± 48 (14–50)	39 ± 41 (16–54)	40 ± 50 (13–49)	.93
Sodium, mmol/L	134 ± 5 (132–137)	134 ± 8 (130–135)	134 ± 4 (132–137)	.56
Concomitant organism isolated				
Bacteria	24 (22)	3 (14)	21 (25)	.392
Respiratory virus	23 (22)	1 (5)	22 (26)	.039
Fungus	8 (7)	2 (9)	6 (7)	.667
Mycobacterium	13 (12)	1 (5)	12 (14)	.296
Antimicrobial therapy	105 (98)	22 (100)	83 (98)^∗^	
Fluroquinolone	102 (95)	22 (100)	80 (94)	.58
Macrolide	3 (3)	0 (0)	3 (4)	1.00
In-hospital mortality	14 (13)	4 (28)	10 (12)	.49

Values are presented as numbers (%) or mean ± standard deviation (interquartile range). ALT = alanine transferase, AST = aspartate transaminase, BUN = blood urea nitrogen, CRP = C-reactive protein, PCR = polymerase chain reaction, PSI = pneumonia severity index, WBC = white blood cells.

∗ One patient with cardiopulmonary arrest was resuscitated, but died due to severe cerebral and cardiac dysfunction in lacks of definite antibiotic treatment for Legionnaires disease. The other patient was admitted for community acquired pneumonia and treated by intravenous ampicillin-sulbactam for 7 days and was discharged with clinical improvement. Eventually, *Legionella* PCR from her sputum revealed positive results after the discharge.

**Table 2:** Radiographic changes in patients with Legionnaires disease who showed initial clinical improvement, compared with initial radiological findings.

	Treatment day 7 (n = 107)	Treatment day 14 (n = 89)	At discharge (n = 30)
Progression of disease	22 (21)	8 (9)	2 (7)
Stable response	43 (40)	34 (38)	9 (30)
Response	42 (39)	47 (53)	19 (63)
Partial response	42 (39)	44 (49)	16 (53)
Complete response	0 (0)	3 (3)	3 (10)

Values are presented as numbers of patients (%).

**Table 3:** Univariable and multivariable analysis of risk factors for radiological deterioration with initial clinical improvement in the patients with Legionnaires disease.

	Univariable analysis	Multivariable analysis	
Variable	OR (95% CI)	Adjusted OR (95% CI)	*P* value
Age, median, yrs	1.09 (0.30–3.96)	–	–
Male sex	1.01 (0.96–1.06)	–	–
PSI	1.02 (1.00–1.04)	1.02 (1.00–1.04)	.036
Hematologic malignancy	1.22 (0.23–6.63)	–	–
Solid tumor	0.96 (0.24–3.88)	–	–
ESRD	0.13 (0.10–1.54)	–	–
Diabetes mellitus	1.05 (0.23–6.63)	–	–
COPD	0.73 (0.12–4.31)	–	–
Mechanical ventilation	3.76 (1.11–12.77)	3.65 (1.15–11.63)	.029

CI = confidence interval, COPD = chronic obstructive pulmonary disease, ESRD = end stage renal disease, OR = odds ratio, PSI = pneumonia severity index score.

## 4 Discussion

In this retrospective study of 107 patients with Legionnaires disease, about one-fifth of all patients, especially those who had high PSI scores and underwent mechanical ventilation, showed radiological deterioration despite clinical improvement 1 week after appropriate treatment. We found a higher mortality rate (28%) in patients with initial clinical improvement who showed radiological deterioration than in those with initial clinical improvement and radiologically stable, partial, or complete responses (12%); the difference did, however, not reach statistical significance. Our findings provide useful information on the management of patients with Legionnaires disease, for example regarding the decision to switch antibiotics.

Several studies have addressed the radiological appearance of Legionnaires disease.^[5–7,16–18]^ However, to our knowledge, the number of studies reporting on the correlation between the radiological and clinical course in Legionnaires disease is limited. Rates of radiographic deterioration in Legionnaires disease have been reported at 58% from day 3 to 6 and 19% after day 6 by Tan et al^[5]^ and at 29% on day 10 by Domingo et al.^[16]^ However, these studies neither depicted the detailed clinical course nor reported correlations with radiological findings. Other studies only reported the patients’ radiological evolution but did not assess clinical correlations during treatment for Legionnaires disease.^[6,7,17,18]^ We found that about one-fifth of patients who experienced clinical improvement showed radiological deterioration, a phenomenon called “clinicoradiological dissociation.” The reasons for this paradoxical radiological worsening are unknown. *L. pneumophilia* is a facultative intracellular pathogen that enters and multiplies in human alveolar macrophages. It has been reported that quinolones have intracellular concentration-dependent antibacterial activity against *L. pneumophilia*, and it takes several days to attain their maximal effective concentration.^[19]^ The intracellular bacteria might continue their multiplication without a satisfactory antibiotic concentration during the early treatment phase. Following intracellular multiplication, inflammatory cells infiltrate the alveoli, capillary leakage results in edema, and cytokines released by infected macrophages help trigger the severe inflammatory response.^[20]^ Alternatively, antibiotic treatment might result in the further release of intracellular antigens due to infected cell death. Patients with high PSI scores and in need of mechanical ventilation are defined as having severe Legionnaires disease, and severe inflammatory reactions in these patients might present as radiological deteriorations despite clinical improvement. In this context, our data warrant further studies on the underlying pathophysiologic mechanisms and the development of new treatment for these patients.

Our study also revealed a higher mortality rate in patients with radiological deterioration than in those without radiological progression, although the difference was not statistically significant. While it is difficult to draw a firm conclusion, owing to the small sample size of the current study, this finding might stem from a lag in radiographic changes or from paradoxical worsening underlying the clinical improvement; it does not necessarily indicate the failure of the antibiotic therapy. Critically ill patients with Legionnaires disease who receive quinolone therapy are frequently switched to other antibiotics or to a broad-spectrum antibiotic therapy due to their initial radiological progression. However, our findings support that close monitoring without modification of antibiotics use is warranted in those who have clinical improvement regardless of radiologic findings. Furthermore, the current tests for the diagnosis of Legionnaires disease have suboptimal sensitivities, so the substantial patients without documentation of Legionnaires disease might receive unnecessary broad-spectrum antibiotics due to radiologic progression despite of quinolone therapy. Therefore, our study raises questions regarding this practice in terms of antibiotic stewardship. Further studies are needed to follow up on these questions.

Our study has several limitations. First, as it was performed at a single center, the number of patients was relatively small to reach any statistical significance for many differences. It is, however, the largest systematic study on the clinical and radiological findings in patients with Legionnaires disease to date. Second, considering the retrospective nature of the study, we could not determine the exact peak time and the precise total resolution because radiographs were obtained at various times during each patient's convalescence period. Third, infections due to *Legionella*, especially non-pneumophilia types, might be underdiagnosed and overlooked in mild illness. *Legionella* species do not grow on most standard media used for primary isolation of respiratory pathogens in the microbiology laboratory. Furthermore, the *Legionella* urinary antigen test that is widely used as a diagnostic test for *Legionella* infection does not reliably detect infection due to *Legionella* species other than *L. pneumophilia* serogroup 1. However, *L. pneumophilia* is the predominant cause of Legionnaires disease, accounting for 90% or more of cases. Additionally, the test might not yield distinct results because clinical and radiographic manifestations of non-pneumophilia *Legionella* species are known to be similar to those of *L. pneumophilia*.^[21]^

In conclusion, patients with Legionnaires disease appear to frequently present with clinicoradiological dissociation during the first week of treatment. However, this radiological worsening is not associated with worse outcomes. Therefore, close monitoring without modification of antibiotics use is warranted in those who have clinical improvement regardless of radiologic findings.

## Author contributions

**Conceptualization:** Sung-Han Kim.

**Data curation:** Jinyeong Kim.

**Formal analysis:** Jinyeong Kim.

**Funding acquisition:** Sung-Han Kim.

**Investigation:** Jinyeong Kim.

**Methodology:** Sung-Han Kim.

**Project administration:** Sunghee Park, Eunmi Yang, Haein Kim, Hyeonji Seo, Hyemin Chung, Jiwon Jung, Min Jae Kim, Yong Pil chong, Sang-Oh Lee, Sang-Ho Choi, Yang Soo Kim, Sung-Han Kim.

**Supervision:** Sung-Han Kim.

**Validation:** Sung-Han Kim.

**Writing – original draft:** Jinyeong Kim.

**Writing – review & editing:** Sung-Han Kim.

## References

[1] Phin N Parry-Ford F Harrison T. Epidemiology and clinical management of Legionnaires’ disease. Lancet Infect Dis 2014;14:1011–21. 24970283 10.1016/S1473-3099(14)70713-3

[2] Fields BS Benson RF Besser RE. Legionella and Legionnaires’ disease: 25 years of investigation. Clin Microbiol Rev 2002;15:506–26. 12097254 10.1128/CMR.15.3.506-526.2002 PMC118082

[3] Beauté J. The European Legionnaires’ Disease Surveillance Network. Legionnaires’ disease in Europe, 2011 to 2015. Euro Surveill 2017;22:30566. 28703097 10.2807/1560-7917.ES.2017.22.27.30566 PMC5508329

[4] Shah P Barskey A Binder A. Legionnaires’ Disease Surveillance Summary Report, United States 2014–2015. Centers for Disease Control and Prevention last updated: 2019.

[5] Tan MJ Tan JS File TM Jr Hamor RH Breiman RF. The radiologic manifestations of Legionnaire's disease. Chest 2000;117:398–403. 10669681 10.1378/chest.117.2.398

[6] Kirby BD Peck H Meyer RD. Radiographic features of Legionnaires disease. Chest 1979;76:562–5. 498829 10.1378/chest.76.5.562

[7] Macfarlane JT Miller AC Roderick Smith WH Morris AH Rose DH. Comparative radiographic features of community acquired Legionnaires’ disease, pneumococcal pneumonia, mycoplasma pneumonia, and psittacosis. Thorax 1984;39:28–33. 6695350 10.1136/thx.39.1.28 PMC459717

[8] Halm EA Fine MJ Kapoor WN Singer DE Marrie TJ Siu AL. Instability on hospital discharge and the risk of adverse outcomes in patients with pneumonia. Arch Intern Med 2002;162:1278–84. 12038946 10.1001/archinte.162.11.1278

[9] Woodhead M Blasi F Ewig S. Guidelines for the management of adult lower respiratory tract infections. Clin Microbiol Infect 2011;17:E1–59. 10.1111/j.1469-0691.2011.03672.x PMC7128977 21951385

[10] Lim WS Baudouin SV George RC. BTS guidelines for the management of community acquired pneumonia in adults: update 2009. Thorax 2009;64:iii1–55. 19783532 10.1136/thx.2009.121434

[11] Ruiz-González A Falguera M Porcel JM. C-reactive protein for discriminating treatment failure from slow responding pneumonia. Eur J Intern Med 2010;21:548–52. 21111942 10.1016/j.ejim.2010.09.006

[12] Fine MJ Auble TE Yealy DM. A prediction rule to identify low-risk patients with community-acquired pneumonia. N Engl J Med 1997;336:243–50. 8995086 10.1056/NEJM199701233360402

[13] Hansell DM Bankier AA MacMahon H McLoud TC Müller NL Remy J. Fleischner Society: glossary of terms for thoracic imaging. Radiology 2008;246:697–722. 18195376 10.1148/radiol.2462070712

[14] Segal BH Herbrecht R Stevens DA. Defining responses to therapy and study outcomes in clinical trials of invasive fungal diseases: Mycoses Study Group and European Organization for Research and Treatment of Cancer consensus criteria. Clin Infect Dis 2008;47:674–83. 18637757 10.1086/590566 PMC2671230

[15] Jung J Hong HL Lee SO. Immune reconstitution inflammatory syndrome in neutropenic patients with invasive pulmonary aspergillosis. J Infect 2015;70:659–67. 25597823 10.1016/j.jinf.2014.12.020

[16] Domingo C Roig J Planas F Bechini J Tenesa M Morera J. Radiographic appearance of nosocomial Legionnaires’ disease after erythromycin treatment. Thorax 1991;46:663–6. 1948796 10.1136/thx.46.9.663 PMC463364

[17] Kroboth FJ Yu VL Reddy SC Yu AC. Clinicoradiographic correlation with the extent of Legionnaire disease. Am J Roentgenol 1983;141:263–8. 6603115 10.2214/ajr.141.2.263

[18] Dietrich PA Johnson RD Fairbank JT Walke JS. The chest radiograph in Legionnaires’ disease. Radiology 1978;127:577–82. 663140 10.1148/127.3.577

[19] Baltch AL Bopp LH Smith RP Michelsen PB Ritz WJ. Antibacterial activities of gemifloxacin, levofloxacin, gatifloxacin, moxifloxacin and erythromycin against intracellular Legionella pneumophila and Legionella micdadei in human monocytes. J Antimicrob Chemother 2005;56:104–9. 15941776 10.1093/jac/dki186

[20] Bennett JE Blaser MJ Dolin R. Mandell, Douglas, and Bennett's Principles and Practice of Infectious Diseases. Vol 2. Philadelphia: Elsevier; 2020.

[21] Muder RR Yu VL. Infection due to Legionella species other than L. pneumophila. Clin Infect Dis 2002;35:990–8. 12355387 10.1086/342884

